# Identification and characterization of alkaline serine protease from goat skin surface metagenome

**DOI:** 10.1186/2191-0855-1-3

**Published:** 2011-03-28

**Authors:** Paul Lavanya Pushpam, Thangamani Rajesh, Paramasamy Gunasekaran

**Affiliations:** 1 Department of Genetics, Centre for Excellence in Genomic Sciences, School of Biological Sciences, Madurai Kamaraj University, Madurai, India 625021; 2 Department of Microbial Engineering, College of Engineering, Konkuk University, 1 Hwanyang-dong, Gwangjin-gu, Seoul 143-701, Korea

**Keywords:** goat skin, metagenome, metagenomic library, serine protease, alkaline protease

## Abstract

Metagenomic DNA isolated from goat skin surface was used to construct plasmid DNA library in *Escherichia coli *DH10B. Recombinant clones were screened for functional protease activity on skim milk agar plates. Upon screening 70,000 clones, a clone carrying recombinant plasmid pSP1 exhibited protease activity. *In vitro *transposon mutagenesis and sequencing of the insert DNA in this clone revealed an ORF of 1890 bp encoding a protein with 630 amino acids which showed significant sequence homology to the peptidase S8 and S53 subtilisin kexin sedolisin of *Shewanella *sp. This ORF was cloned in pET30b and expressed in *E. coli *BL21 (DE3). Although the cloned Alkaline Serine protease (AS-protease) was overexpressed, it was inactive as a result of forming inclusion bodies. After solubilisation, the protease was purified using Ni-NTA chromatography and then refolded properly to retain protease activity. The purified AS-protease with a molecular mass of ~63 kDa required a divalent cation (Co^2+ ^or Mn^2+^) for its improved activity. The pH and temperature optima for this protease were 10.5 and 42°C respectively.

## Introduction

Proteases are present in all living forms as they are involved in various metabolic processes. They are mainly involved in hydrolysis of the peptide bonds (Gupta et al. 2002). Proteases are classified into six types based on the functional groups in their active sites. They are aspartic, cysteine, glutamic, metallo, serine, and threonine proteases. They are also classified as exo-peptidases and endo-peptidases, based on the position of the peptide bond cleavage. Proteases find a wide range of applications in food, pharmaceutical, leather and textile, detergent, diagnostics industries and also in waste management (Rao et al. 1998). Thus, they contribute to almost 40% of enzyme sales in the industrial market. Though proteases are found in plants and animals, microbial proteases account for two-third of share in the commercially available proteases (Kumar and Takagi 1999).

Proteases are also classified as acidic, neutral or alkaline proteases based on their pH optima. The largest share of the enzyme market is occupied by detergent proteases, which are mostly alkaline serine protease and active at neutral to alkaline pH range. Alkaline serine proteases have Aspartate (D) and Histidine (H) residues along with Serine (S) in their active site forming a catalytic triad (Gupta et al. 2002). Serine proteases contribute to one third of the share in the enzyme market and are readily inactivated by Phenyl Methane Sulfonyl Fluoride (PMSF) (Page and Di Cera 2008). Based on the sequence and structural similarities, all the known proteases are classified into clans and families and are available in the MEROPS database (Rawlings and Barrett 1993).

Several microbial proteases from the culturable organisms have been characterized. However, very few proteases have been identified through culture independent metagenomic approach (Schloss and Handelsman 2003). In metagenomics study, the total nucleic acid content of the environmental samples is analysed. The DNA may be isolated by direct or indirect methods followed by purification (Gabor et al. 2003); Rajendhran and Gunasekaran 2008). Metagenomics approach has been recently employed in identifying number of novel genes encoding biocatalysts or molecules which are of pharmaceutical and industrial importance. Interestingly, the metagenomic libraries were mainly screened for enzymes like lipases and esterases (Lee et al. 2004; Rhee et al. 2005; Voget et al. 2003), proteases (Lee et al. 2007), amylases (Rondon et al. 2000; Voget et al. 2003), chitinase (Cottrell et al. 1999) and nitrilases (Robertson et al. 2004). Despite the success rate, very few attempts were made on the identification of proteases from metagenomic libraries. We report here an Alkaline Serine protease (AS-protease), identified from the goat skin metagenomic library, which showed homology to peptidase S8 and S53 subtilisin kexin and sedolisin of *Shewanella *sp. Surprisingly, this AS-protease requires Co^2+ ^or Mn^2+ ^metal ions for its improved activity.

## Materials and methods

### Materials, bacterial strains and culture conditions

Goat skins were obtained from butcheries in and around Madurai for metagenomic DNA isolation. Reagents for PCR, *Taq *DNA polymerase, oligonucleotide primers, and all biochemicals were from Sigma-Aldrich (St. Louis, MO, USA). T4 DNA ligase and restriction enzymes were from MBI Fermentas (Opelstrasse, Germany). *Escherichia coli *strains and plasmids used in this study are listed in Table 1. *E. coli *DH5α and *E. coli *BL21 (DE3) were used for gene cloning and protein expression studies respectively.

**Table 1:** List of bacterial strains and plasmids used in this study

Strains/plasmids	Genotype/Description	Reference/Source
*E. coli *DH5α	F^- ^*end*A1 *gln*V44 *thi-*1 *rec*A1 *rel*A1 *gyr*A96 *deo*R *nup*G *Φ80dlac*ZΔ*M15 *Δ*(lac*ZYA*-arg*F*) *U169*, hsd*R17*(r_K_^- ^m_K_^+^), λ-*	Invitrogen (CA, USA)
*E. coli *DH10B	F^- ^*end*A1 *rec*A1 *gal*E15 *gal*K16 *nupG rps*L Δ*lac*X74 *Φ80lac*ZΔM15 *ara*D139 Δ*(ara, leu)*7697 *mcr*A Δ*(mrr-hsd*RMS-*mcr*BC*) λ^-^*	Invitrogen (CA, USA)
*E. coli *BL21 (DE3)	F^- ^*omp*T *gal dcm lon hsd*S_B_*(r_B_^- ^m_B_^-^) λ(*DE3 *[lacI lac*UV5-T7 *gene*1 *ind*1 *sam*7 *nin*5*])*	Novagen (CA, USA)
		
pUC19	*Ap^r^; *Cloning vector	Stratagene (CA, USA)
pTZ57R/T	*Ap^r^*; PCR cloning vector	MBI Fermentas (Opelstrasse, Germany)
pET30b	*Kn^r^*; Expression vector; T7 promoter	Novagen (CA, USA)
pSP1	pUC19 harbouring the AS-protease ORF; *Ap^r^*	This study
pTSP1	AS- Protease ORF cloned in pTZ57R/T; *Ap^r^*	This study
pETP1	AS- protease ORF cloned in pET30b; *Kn^r^*	This study

### DNA manipulation techniques

Standard procedures for plasmid isolation, restriction enzyme digestion, ligation, competent cell preparation and transformation were used as described by (Sambrook et al., (1989)). Metagenomic DNA was isolated using a modified indirect DNA extraction method (Gabor et al. 2003). The goat skin (10 cm × 10 cm) was suspended in 0.75% (w/v) NaCl and kept under agitation at 180 rpm for 30 min. The supernatant was collected and a pellet was obtained by centrifugation (10,000 × g for 10 min at 4°C). The pellet was rinsed and suspended in blending buffer (100 mM Tris-HCl [pH 8.0], 100 mM sodium EDTA [pH 8.0], 0.1% SDS) and homogenized. The homogenized mixture was subjected to low-speed centrifugation (1000 × *g *for 10 min at 10°C), and the supernatant containing bacterial cells was collected, while the coarse particles and high molecular weight DNA in the pellet was subjected to further centrifugation cycles as described above. Supernatant obtained from the three rounds of cell extraction were pooled. The supernatant were centrifuged at 10,000 × *g *for 30 min at 4°C and the cell pellet was rinsed with chrombach buffer (0.33 M Tris-HCl, 1 mM EDTA, pH 8). Then the mixture was suspended in lysis buffer (100 mM Tris-HCl, 100 mM EDTA, 1.5 M NaCl), in the presence of 0.1 mg of proteinase K and 1 mg of lysozyme and incubated at 37°C for 30 min. Lysis was completed by adding 1 ml of 20% SDS and incubated for 2 h at 65°C with shaking every 30 min. The supernatant was collected by centrifugation at 6000 × *g *for 10 min at 30°C and the pellets were re-extracted twice with 1 ml lysis buffer, vortexing for a few seconds, and incubating at 65°C for 10 min. The supernatant was extracted with equal volume of chloroform: isoamyl alcohol (24:1). DNA in the aqueous phase was precipitated by addition of 0.6 volumes of isopropanol and incubated at -20°C for 1 h. The precipitate was collected by centrifugation at 10,000 × g for 15 min at 4°C and then washed with 70% ethanol. The DNA pellet was suspended in 200 μl TE buffer (10 mM Tris-HCl, 1 mM EDTA, pH 8) and stored at -20°C.

Metagenomic DNA was partially digested with *Hind*III and the DNA fragments ranging about 3-8 kb were separated with QIAquick gel extraction kit (Qiagen, Hilden, Germany) and cloned into pUC19, resulting in plasmid pSP1 which was transformed into *E. coli *DH10B by electroporation (200 Ω, 25 μF and 2.5 kV) using Gene Pulser (Bio-Rad, USA). Transformants were selected on LB agar plates supplemented with 100 μg of ampicillin/ml, X-gal (20 μg/ml) and IPTG (40 μg/ml) and incubated at 37°C for overnight. The white recombinant clones were scored and maintained.

### Screening the metagenomic library for proteolytic activity

The recombinant clones were screened for proteolytic activity on LB agar ampicillin plates supplemented with 1% (w/v) skim milk (Lee et al. 2007) and incubated at 37°C for 48 - 72 h. Proteolytic clones were selected based on the formation of zone of clearance around the colony.

### *In vitro *transposon mutagenesis and sequencing

The recombinant plasmid was used as template for *in vitro *transposon mutagenesis using Template Generation System II kit (TGS, F-702; Finnzyme, Finland). *E. coli *DH10B carrying the plasmid pSP1 was transformed with the artificial Mu transposon by electroporation and the transformants were selected on LB agar plates containing ampicillin (100 μg/ml) and kanamycin (30 μg/ml). Further, the strains carrying the plasmid with the mutated protease were screened on 1% skim milk-LB agar plate for a negative activity. The plasmids from the mutants were isolated and the regions adjacent to the transposons were sequenced using transposon specific primer. BlastN and BlastP analyses were carried out to find sequence identity and homology (Altschul et al. 1990). Signal peptide of the protein was predicted using the SignalP 3.0 server http://www.cbs.dtu.dk/services/SignalP/ (Bendtsen et al. 2004). Multiple sequence alignment was performed with the sequences (MER048892; *Shewanella baltica*, MER087187; *Shewanella woodyi*, MER016525; *Pseudoalteromonas sp*. AS-11) in the MEROPS peptidase database http://merops.sanger.ac.uk (Rawlings and Barrett 1993) to assign the family for the identified protease and also in the NCBI database.

### Cloning and expression of protease encoding gene

The complete ORF of the protease was amplified with the primers MP1F (5'-ATGCATAAGAAACATTTAATAGCA3') and MP1R (5'CTAGTAGCTTGCACTCAGCTGAAC-3') and cloned into pTZ57R/T vector, and the resultant plasmid was used to transform *E. coli *DH5α. The cloned protease gene was confirmed by DNA sequencing using the BigDye Terminator sequencing method and an ABI PRISM 3700 sequencer (Applied Biosystems, Foster City, CA). The protease gene was again amplified from the recombinant plasmid with and without the signal peptide using forward primers P1FS 5' - GCGCCATATGCATAAGAAACATTTAATAG-3' (*Nde*I site is underlined) and P1FWS 5'- ATTACATATGGAATACCAAGCGACTATGGTAAG-3' (*Nde*I site is underlined) and reverse primer P1RH 5'-TAATAAGCTTGTAGCTTGCACTCAGCTG-3' (*Hind*III site is underlined). The PCR product was digested with *Nde*I and *Hind*III and ligated with expression vector pET30b to obtain another recombinant plasmid, in which the protease gene was under the control of the T7 promoter. This recombinant plasmid was then used to transform *E. coli *BL21 (DE3). *E. coli *BL21 (DE3) carrying recombinant plasmid was grown overnight at 37°C in LB medium containing kanamycin (30 μg/ml). Fresh LB medium with kanamycin was inoculated with 1% (v/v) of overnight culture and incubated at 37°C until the culture reached an absorbance of 0.4 at OD_600_. The culture was then induced with 0.1 mM of isopropyl-β-D-thiogalactopyranoside (IPTG). The induced cells were harvested by centrifugation at 4°C for 10 min at 12,000 × g and washed with 50 mM Tris-buffer (pH 7.5). The cells were then disrupted by sonication (five times for 30 s with 30 s interval) (Labsonic U, Germany), and centrifuged at 12 000 × *g *for 30 min. Both the soluble and pellet fractions were analysed for protease activity.

### SDS-PAGE and Zymogram analysis

The proteins from the insoluble fraction after sonication were resolved on Sodium dodecyl sulphate-polyacrylamide gel electrophoresis (SDS-PAGE) (Laemmli 1970). The gel was stained with Coomassie brilliant blue R-250. The molecular mass of protein was determined by comparison with the mobility of molecular weight markers (Fermentas, Opelstrasse, Germany). For zymogram analysis, the protein were separated on the SDS-PAGE with 0.1% (w/v) gelatin in the separating gel (Bressollier et al. 1999). After electrophoresis, the gel was incubated with 2.5% (v/v) Triton X-100 at 37°C for 30 min for the removal of SDS followed by another round of incubation in 50 mM Tris (pH 7.4) for 30 min. The gel was then incubated in the same buffer at 37°C for 4 h. Zone of clearance within the gel was checked after staining with Coomassie brilliant blue R-250.

### Purification of protease

The cell pellets was resuspended in 20 mM Tris-HCl buffer (pH 7.5), disrupted by sonication and centrifuged at 10,000 × g for 30 min. The insoluble fraction after sonication, containing the recombinant protein was collected and solubilised in 3 ml of cold 2 M urea containing 20 mM Tris-HCl buffer, 0.5 M NaCl and 2% Triton X-100 (pH 8.0) and centrifuged at 10,000 × g for 10 min. The supernatant was discarded and the pellet fraction was further washed once with the same buffer and then resuspended in 5 ml of 20 mM Tris-HCl buffer containing 8 M urea, 0.5 M NaCl, 5 mM imidazole, 1 mM 2-mercaptoethanol (pH 8.0), and stirred at room temperature for 30-60 min to solubilise the recombinant protein. The solubilised proteins were passed through Ni-NTA Affinity column (Sigma Chemicals, USA) and eluted with imidazole following the manufacturer's recommendation. The purified protein with urea was then refolded in 20 mM Tris buffer by drop dilution method (Howarth et al. 2006). The refolded protein was used for further characterization.

### Enzyme assay

In standard conditions, the reaction mixture contained 480 μl of 1% (w⁄ v) azocasein, 2 mM CaCl_2 _and appropriate dilution of enzyme in 50 mM Tris buffer, pH 7.5 (Radha and Gunasekaran 2007). The reaction mixture was incubated at 37°C for 30 min. The reaction was terminated by adding 600 μl of 10% (w/v) trichloroacetic acid and kept on ice for 15 min followed by centrifugation at 15,000 × g at 4°C for 10 min. Eight hundred microlitre of the supernatant were neutralized by adding 200 μl of 1.8 N NaOH, and the absorbance at 420 nm (A_420_) was measured using a spectrophotometer (Hitachi U-2000, Japan). The control samples were the extract from the *E. coli *BL21 (pET30b) only. One unit of protease activity was defined as the amount of enzyme required to yield an increase in absorbance of 0.01 at A_420 _in 30 min at 37°C.

### Effect of metal ions, inhibitors, solvents, detergents and reducing agents

Protease was purified as previously described followed by extensive dialysis in the presence of 10 mM EDTA in 50 mM Tris buffer (pH 7.5) and then, the enzyme was assayed under standard conditions in the presence of different metal ions (Mn^2+^, Ca^2+^, Co^2+^, Ni^2+^, Hg^2+^and Zn^2+^). The purified protease was pre-incubated with different metal ions (0.1, 1 and 5 mM), inhibitors (5 mM), detergents (0.5 - 1%) and reducing agent (β-ME) (5 mM) for 15 min at 37°C. The residual activity was measured under standard assay condition.

### Physicochemical characterization

The effect of temperature on the activity of the purified AS-protease was determined at the temperature range of 10°C to 85°C at pH 7.5. Thermal stability of the purified AS-protease was estimated by incubating the enzyme in 50 mM Tris buffer at different temperatures (35°C, 45°C and 55°C) in the presence of 5 mM CoCl_2_. At different intervals, samples were withdrawn and the residual activity was measured under standard assay condition. The optimum pH of AS-protease activity was measured at 37°C with different buffer: 50 mM Sodium acetate buffer (pH 4-5.5), 50 mM Tris buffer (pH 6.5-8.5), 50 mM sodium carbonate buffer (pH 9), and 50 mM glycine-NaOH buffer (pH 10.5-12.5).

### Determination of kinetic parameters

The recombinant protease was assayed with 0.1-10 mg/ml azocasein in 50 mM Tris buffer (pH 7.5) containing 5 mM Co^2+ ^at 42°C for 10 min. Kinetic parameters, such as K_m _(mg/ml) *K*_cat _(min^-1^) and V_max _(U/mg-protein) for substrates were obtained using Line-weaver Burk plot.

## Results

### Construction and screening of metagenomic library from Goat skin

Diverse microbial population (both culturable and non culturable) with majority of them with proteolytic activity was found on the goat skin surface (Kayalvizhi and Gunasekaran, 2008). Therefore, metagenomic DNA (~5 μg/ml) of the goat skin surface was isolated by an indirect extraction method as described in materials and methods. A small-insert metagenomic library in pUC19 was constructed. Analysis of the randomly selected recombinant clones revealed that the clones had the insert DNA of an average size of ~3.2 kb.

**Figure 1:**
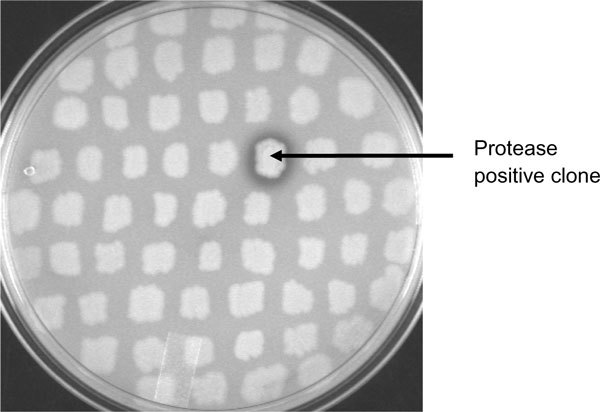
**Functional screening of metagenomic library for protease activity on skim milk agar plate**. Metagenomic library consisting of 70,000 clones were screened on skim milk plate for protease activity. The positive clone showing zone of clearance in skim milk agar plate is indicated by an arrow.

**Figure 2:**
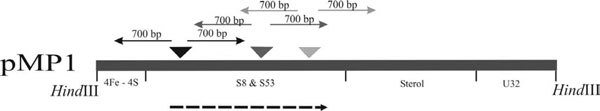
**Schematic representation of the insert metagenomic DNA and the position of transposon used for sequencing the coding region**. Each inverted triangle represents the individual insertion of transposon in the protease coding gene. Black dotted arrow indicates the orientation and location of protease gene. 4Fe-4S represents 4Fe-4S ferredoxin iron-sulfur binding domain protein, S8 & S53 - peptidase S8 and S53 subtilisin kexin sedolisin, sterol - Sterol-binding domain protein, U32 - peptidase U32.

**Figure 3:**
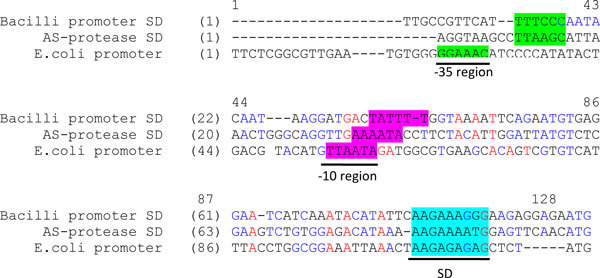
**Comparison of AS-protease promoter with other promoter sequences**. A probable promoter regions (-35, -10 region) and a Shine-Dalgarno (SD) region is shown by solid lines and is highlighted. *Bacilli *protease promoter represents, *Bacillus stearothermophilus *protease promoter. Protease promoter represents the predicted alkaline serine protease promoter region. *E. coli *protease promoter represents, *E.coli *lon protease promoter.

Screening of 70,000 recombinant clones for proteolytic activity revealed one clone carrying recombinant plasmid designated as pSP1 that exhibited a zone of clearance on LB skim milk agar plate after 36 h of incubation at 37°C (Figure 1). Since insert DNA in this clone was 3.8 kb (Figure 2), the protease gene could have been expressed with its own promoter (Figure 3). Transposon mutagenesis on pSP1 was carried out to have Tn insertion within the protease coding region in the insert DNA (Figure 2). Randomly selected transposon carrying protease negative mutants were sequenced and alignment of these sequences lead to the identification of the protease open reading frame (ORF).

### Analysis of the cloned protease gene

**Figure 4:**
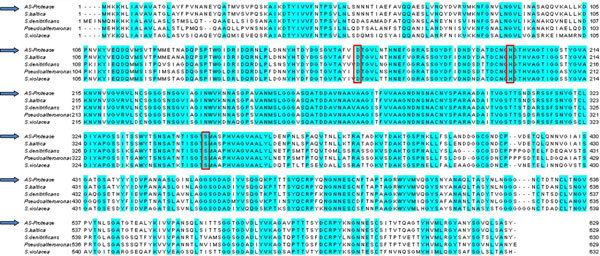
**Multiple sequence alignment of AS-protease gene sequence from metagenome**. Proteases used for alignment are *S. baltica*, peptidase S8 and S53 subtilisin kexin sedolisin [*Shewanella baltica *OS185] (YP_001367387.1)*; S. violacea*, extracellular alkaline serine protease precursor, putative [*Shewanella violacea DSS12*] (YP_003556880.1)*; S. denitrificans*, peptidase S8 and S53, subtilisin, kexin, sedolisin [*Shewanella denitrificans OS217*] (YP_562027.1). *Pseudoalteromonas*, extracellular alkaline serine protease 2 [*Pseudoalteromonas sp*. AS-11]. The AS-protease sequence identified from metagenome is indicated by arrows in the left. Conserved residues are letters in dark blue background. Catalytic residues are boxed in red outline.

The ORF encoding the protease was amplified and cloned in pTZ57R/T vector and the resultant construct was designated as pTSP1. Analysis of the insert DNA sequence as described above, revealed an ORF (1890 bp) with ATG as start codon and TAG as termination codon. The deduced amino acid sequence of the protease comprises of 630 amino acids and an estimated molecular mass of 65,540 Da. Multiple sequence alignment of this protease was performed with other known protease sequences in the NCBI database and shown in Figure 4. The amino acid sequence of this AS-protease displayed 98% sequence similarity with uncharacterized proteases of various *Shewanella *sp. in the NCBI database and a maximum of 85% similarity with S8A secreted peptidaseA of *Shewanella baltica *MEROPS database (Rawlings and Barrett 1993). These results suggested that the cloned protease belongs to serine family peptidase.

At the N terminus of this AS-protease sequence, presence of a signal peptide with 23 amino acids was predicted using the SignalP program (Bendtsen et al. 2004). The Pfam analysis of this protease showed a conserved catalytic domain of peptidase S8 family and two pre-peptidase C-terminal domains. This AS-protease contained active site residues within the catalytic motif **Asp**-Thr/Ser-Gly, **His**-Gly-Thr-His and Gly-Thr-**Ser**-Met-Ala-X-Pro, which is characteristic of serine subfamily S8A. Results from the sequence analysis of this protease suggested it to be serine protease subfamily S8A.

### Expression of AS-protease gene

The protease coding ORF was amplified and cloned into the expression vector pET30b and resultant recombinant plasmid was designated as pETP1. Upon induction, the *E. coli *BL21 (DE3) harbouring the recombinant plasmid pETP1 expressed the cloned protease gene.

**Figure 5:**
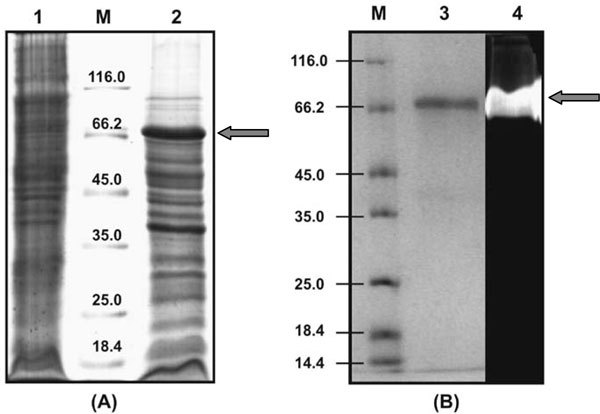
**SDS-PAGE and zymogram analysis of the purified AS-protease**. Lane M, molecular weight marker proteins (14.4 to 116 kDa); Solublised pellet fraction of *E. coli *BL21 (pET30b) (lane 1) and *E. coli *BL21 (pETP1) (lane 2); purified AS-protease (lane 3); zymogram of purified protease (lane 4). An arrow indicates the purified AS- protease.

Further, proteins in the recombinant cell extract was resolved on SDS-PAGE revealed an over expressed protein of 66 kDa (Figure 5A) which is in agreement with the predicted molecular mass for the cloned AS-protease. The protein was expressed as inclusion bodies, which was later solubilised with urea as mentioned in materials and methods. The solubilised protein was purified on Ni-NTA Affinity Chromatography (Figure 5B) and then refolded by drop dilution. The purified refolded protein exhibited a maximum activity of 100.2 U ml^-1 ^(specific activity 83.56 U mg^-1^).

### Effect of pH and temperature

**Figure 6:**
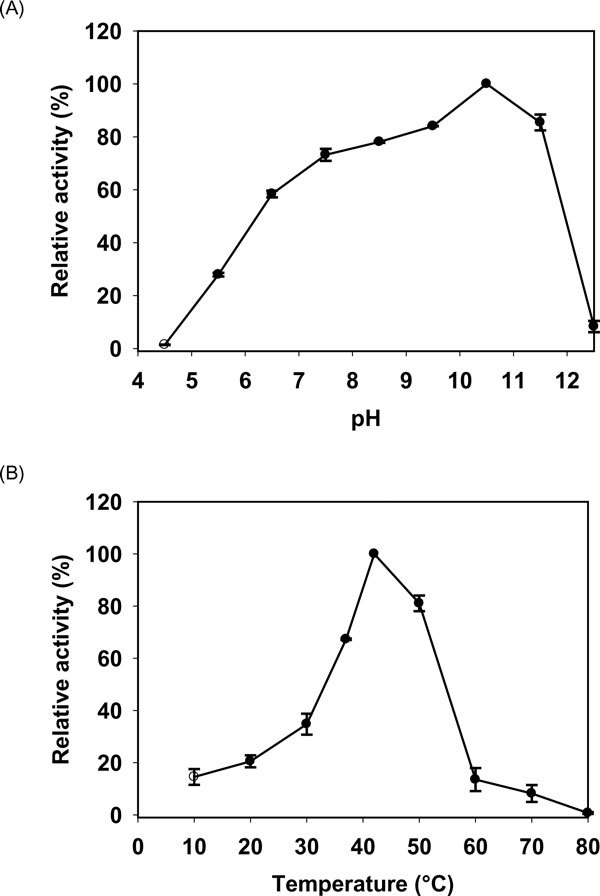
**Effect of pH and temperature on the activity of AS-protease**. The AS- protease activity was maximum at pH 10.5 (A) and at temperature 42°C (B) and these values were taken as 100% for comparison. Each value represents the mean of triplicate measurements and varied from the mean by not more than 10%.

**Figure 7:**
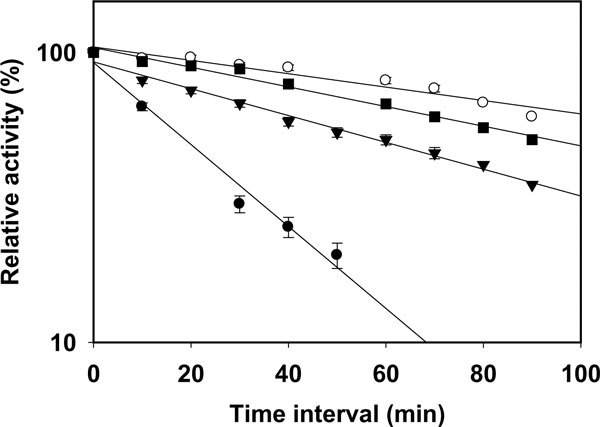
**Thermal stability profiles of the purified protease in the presence of 5 mM Co^2+ ^at 55°C (Black circle), 45°C (Inverted triangle), 40°C (Black Square) and 35°C (Open circle)**. Residual activity was measured at standard conditions.

The effect of pH on the purified AS-protease was examined at 37°C. Purified AS-protease exhibited maximum activity at pH 10.5 (Figure 6A), confirming it to be an alkaline protease. This protease exhibited 75 - 85% of activity at a pH range of 7.5 to 9.5. The proteolytic activity was significantly decreased above pH 11.5 and below pH 7.0. Proteolytic activity was found maximum at 42°C (Figure 6B) but exhibited only 65 and 85% of the maximum activity at the temperature range of 35°C and 55°C respectively. Thermal stability of the purified AS-protease was estimated at different temperatures (35°C, 45°C and 55°C) in the presence of 5 mM CoCl_2 _and activity was measured at 42°C. The AS-protease was stable at 35°C for 60 min. However, the stability of this protease decreased drastically between 45°C and 55°C with half-life of 60 and 20 min respectively (Figure 7).

### Effects of metal ions and additives

The **AS-**protease activity was estimated in the presence of metal ions (5 mM) and different additives. Protease was purified as previously described without metal ions followed by extensive dialysis in the presence of 10 mM EDTA. All metal ions at low concentrations (0.1 mM and 1 mM) did not affect significantly the protease activity. Even at 5 mM concentration, Zn^2+^, Hg^2+ ^and Ni^2+ ^did not affect the protease activity whereas Fe^2+ ^significantly inhibited protease activity. However, Co^2+ ^and Mn^2+ ^enhanced protease activity by 2.25 and 2 fold respectively (Table 2). This improved protease activity was not affected by the presence of EDTA.

**Table 2:** Effect of inhibitors, metal ions and solvents on AS-protease activity.

Additives	Relative activity (%)
None	100
PMSF (5 mM)	22
EDTA (5 mM)	100
DTT (5 mM)	38
β-ME (5 mM)	38
DMSO (1%)	34
SDS (0.5%)	26
Iso-propanol (1%)	125
MnCl_2 _(5 mM)	200
CaCl_2 _(5 mM)	138
CoCl_2 _(5 mM)	225
NiSO_4 _(5 mM)	109
FeSO_4 _(5 mM)	27
HgCl_2 _(5 mM)	113
ZnCl_2 _(5 mM)	94

The purified AS-protease was preincubated with inhibitors, metal ions or additives/solvents for 15 min at 37°C. The activity of protease measured without any additive was set as 100%.

### Substrate specificity

The substrate specificity of AS-protease was examined by using different proteins (Casein, Bovine serine albumin (BSA) and gelatin [0.1% w/v]) as substrate in the reaction mixtures. AS-protease exhibited relatively high activity on casein. But this protease exhibited only 55 and 58% activity on BSA and Gelatin substrates respectively.

### Kinetic parameters

**Figure 8:**
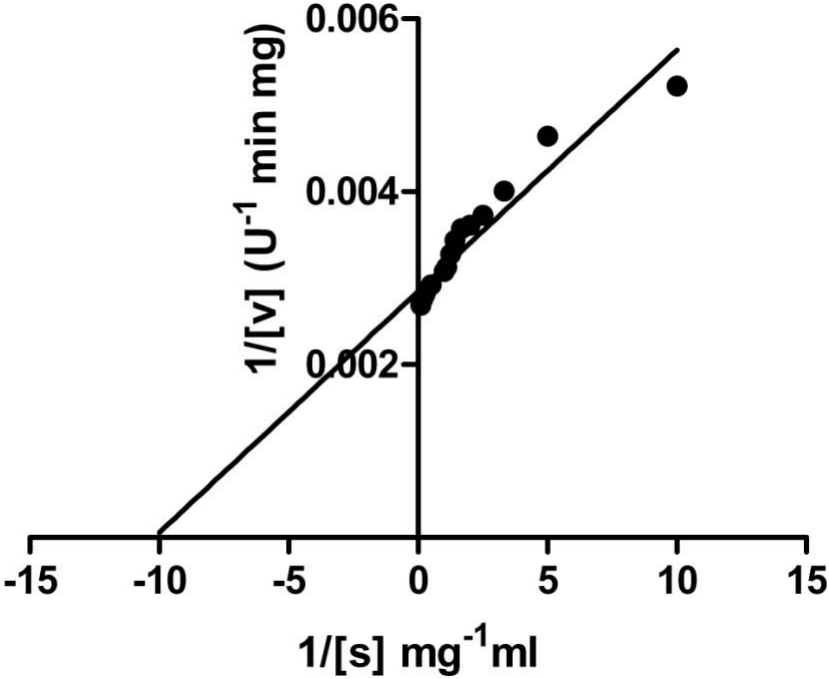
**Lineweaver-Burk plot of the AS- protease**. The Lineweaver-Burk plot was made from the results of the protease assay using different concentrations of azocaesin as substrate under standard conditions. The calculated Vmax and *K*m for azocasein are 366 U/mg and 0.13 mg/ml respectively.

Initial velocities of the purified AS-protease on different concentrations of azocasein were determined under the standard assay conditions at pH 10.5 (Figure 8). The Lineweaver-Burk plot was constructed and the calculated V_max_, *K*_m _and *k*_cat _for azocasein are 366 U/mg, 0.13 mg/ml and 24,156 min^-1 ^respectively.

### Nucleotide sequence accession number

The nucleotide sequence of the AS-protease gene obtained from metagenome was deposited in the GenBank database under the accession number HM370566.

## Discussion

In this study, an attempt was made to identify a protease gene from the goat skin surface metagenome. The eukaryotic DNA concentration was lower in the metagenomic DNA prepared using the indirect methods than the direct method (Gabor et al. 2003). Therefore, we have used indirect extraction method for the isolation of metagenomic DNA from goat skin surface and we were able to identify, overexpress, purify and characterize a protease gene by screening recombinant clones.

We have earlier reported that goat skin contains diverse species of bacteria including several unculturable bacteria in addition to the culturable proteolytic bacteria that are predominant and are involved in the degradation of the skin (Kayalvizhi and Gunasekaran 2008). This does not rule out the possible role of the unculturable bacteria in the degradation of the animal skin. Therefore, the goat skin surface was selected as DNA source for the construction of metagenomic library and to screen for protease gene. Identification of protease gene from metagenomic library was previously unsuccessful (Jones et al. 2007; Rondon et al. 2000). However, few other functional metalloproteases were identified through metagenomic approach (Lee et al. 2007; Waschkowitz et al. 2009; Gupta et al. 2002). The unsuccessful attempts in identification of protease genes from metagenomic library could be attributed to the problems associated with the expression of cloned gene in the heterologous host (Handelsman 2004) and low frequency of target sequence in the metagenomic library (Henne et al. 1999). The serine protease gene identified in the present study showed maximum similarity with peptidase S8 and S53 subtilisin kexin and sedolisin from *S. baltica*. Though the sequence from *S. baltica *is available in the NCBI database, there are no reports on the functional characterization of the peptidase S8 and S53 subtilisin kexin and sedolisin from *S. baltica*. MEROPS database search confirmed that the AS-protease belongs to serine protease S8A family (Jaton-Ogay et al. 1992; Larsen et al. 2006). Based on the multiple sequence alignment, it was found that the catalytic amino acids are conserved as a catalytic triad (D165, H198 and S350) as found in other proteases (Larsen et al. 2006; Rawlings and Barrett 1993).

The metagenome insert sequence was similar to the sequence found in different strains of *Shewanella*, suggesting that the insert from metagenome could have been derived from a strain of *Shewanella *sp. Majority of *Shewanella *sp. are of marine origin (Fredrickson et al. 2008), among which few species are involved in spoilage of fish under stored conditions (Jorgensen and Huss 1989). Thus it is presumed that members of *Shewanella *sp. are present in the microbiome of the goat skin during degradation. Members of *Shewanella *sp. are Gram-negative bacteria belonging to the class *Gammaproteobacteria*. Significant similarity between *Shewanella *and *E. coli *could be responsible for the possible expression of cloned gene heterologous system.

Although AS-protease gene was expressed, this protease was produced as inclusion bodies in *E. coli *when it was overexpressed. Similar expression was seen with subtilisin-like protease gene from *Shewanella *sp. (Kulakova et al. 1999). A lipase gene from a metagenome was also reported to be overexpressed in *E. coli *(Park et al. 2007) and produced as inclusion bodies. In this case, the lipase activity was detected in zymogram. In the present study, the AS- protease in the inclusion bodies was inactive but was solubilised and purified under denaturing conditions. The purified AS-protease was then refolded by drop dilution method to recover its activity. Similarly, cysteine proteinase of *E. histolytica *was recovered from the inclusion bodies (Quintas-Granados et al. 2009).

Alkaline proteases find a number of applications in food industry (Neklyudov et al. 2000), leather processing industry (Varela et al. 1997), waste management (Dalev 1994), medical applications (Kudrya and Simonenko 1994). Proteases are used in detergents and cleaning agent for a long time (Sakiyama et al. 1998; Showell 1999). The purified metagenomic AS-protease showed maximum activity at pH 10.5 suggesting that it is an alkaline protease (Larsen et al. 2006; Moreira 2003). The purified protease was inhibited by phenyl methyl sulfonyl fluride (PMSF), which is a characteristic nature of serine protease (Gupta et al. 2002; Moreira 2003; Xiaoqing Zhang et al. 2010). DTT, β-ME and DMSO were found to inhibit the protease activity, as observed with property of other proteases (Sierecka 1998). In general, most of the serine proteases show enhanced activity in the presence of Ca^2+ ^(Dodia et al. 2008; Singh et al. 2001). In our study, Co^2+ ^and Mn^2+ ^had improved the AS-protease activity by 2.5 and 2 fold respectively. These metal ions may be important cofactors for the proteolytic activity of the enzyme (Ghorbel et al. 2003; Kumar and Takagi 1999).

The largest share of the enzyme market is occupied by detergent resistant proteases which are active and stable in the alkaline pH range (Gupta et al. 2002). The Serine proteases of S8A (subtilisin-like) are generally used in laundry and detergent industries. Hence, the identified AS-protease with maximum activity at alkaline pH range of 10.5 will find application in the detergent and laundry industries. Also metal ions play an important role in enhancing the enzyme activity. According to earlier reports, Ca^2+ ^enhanced the protease activity (Dodia et al. 2008; Singh et al. 2001) and stability. We report here for the first time that Co^2+ ^enhances the protease activity. Hence, AS-protease in the presence of Co^2+ ^can be used in detergent industries.

In summary, functional screening of the metagenomic library revealed a protease positive clone. The sequence analysis and enzyme assay strongly suggested that this alkaline protease is a member of serine protease family. This AS-protease is ready for detailed investigation such as X-ray crystallography and protein engineering studies to understand the molecular mechanism of its activity. Thus, the functional metagenomics pave the way to discover novel genes for biotechnological applications.

## Competing interests

The authors declare that they have no competing interests.
